# Novel roles for well-known players: from tobacco mosaic virus pests to enzymatically active assemblies

**DOI:** 10.3762/bjnano.7.54

**Published:** 2016-04-25

**Authors:** Claudia Koch, Fabian J Eber, Carlos Azucena, Alexander Förste, Stefan Walheim, Thomas Schimmel, Alexander M Bittner, Holger Jeske, Hartmut Gliemann, Sabine Eiben, Fania C Geiger, Christina Wege

**Affiliations:** 1 Institute of Biomaterials and Biomolecular Systems, Department of Molecular Biology and Plant Virology, University of Stuttgart, Pfaffenwaldring 57, Stuttgart, D-70550, Germany; 2 Institute of Functional Interfaces (IFG), Chemistry of Oxidic and Organic Interfaces, Karlsruhe Institute of Technology (KIT), Hermann-von-Helmholtz-Platz 1, Eggenstein-Leopoldshafen, Karlsruhe, D-76344, Germany; 3 Institute of Nanotechnology (INT) and Karlsruhe Institute of Applied Physics (IAP) and Center for Functional Nanostructures (CFN), Karlsruhe Institute of Technology (KIT), INT: Hermann-von-Helmholtz-Platz 1, Eggenstein-Leopoldshafen, D-76344, Germany, and IAP/CFN: Wolfgang-Gaede-Straße 1, Karlsruhe, D-76131 Germany; 4 CIC Nanogune, Tolosa Hiribidea 76, E-20018 Donostia-San Sebastián, Spain, and Ikerbasque, Maria Díaz de Haro 3, E-48013 Bilbao, Spain

**Keywords:** biotemplate, enzyme biosensor, nanotechnology, tobacco mosaic virus, virus-like particles

## Abstract

The rod-shaped nanoparticles of the widespread plant pathogen *tobacco mosaic virus* (TMV) have been a matter of intense debates and cutting-edge research for more than a hundred years. During the late 19th century, their behavior in filtration tests applied to the agent causing the 'plant mosaic disease' eventually led to the discrimination of viruses from bacteria. Thereafter, they promoted the development of biophysical cornerstone techniques such as electron microscopy and ultracentrifugation. Since the 1950s, the robust, helically arranged nucleoprotein complexes consisting of a single RNA and more than 2100 identical coat protein subunits have enabled molecular studies which have pioneered the understanding of viral replication and self-assembly, and elucidated major aspects of virus–host interplay, which can lead to agronomically relevant diseases. However, during the last decades, TMV has acquired a new reputation as a well-defined high-yield nanotemplate with multivalent protein surfaces, allowing for an ordered high-density presentation of multiple active molecules or synthetic compounds. Amino acid side chains exposed on the viral coat may be tailored genetically or biochemically to meet the demands for selective conjugation reactions, or to directly engineer novel functionality on TMV-derived nanosticks. The natural TMV size (length: 300 nm) in combination with functional ligands such as peptides, enzymes, dyes, drugs or inorganic materials is advantageous for applications ranging from biomedical imaging and therapy approaches over surface enlargement of battery electrodes to the immobilization of enzymes. TMV building blocks are also amenable to external control of in vitro assembly and re-organization into technically expedient new shapes or arrays, which bears a unique potential for the development of 'smart' functional 3D structures. Among those, materials designed for enzyme-based biodetection layouts, which are routinely applied, e.g., for monitoring blood sugar concentrations, might profit particularly from the presence of TMV rods: Their surfaces were recently shown to stabilize enzymatic activities upon repeated consecutive uses and over several weeks. This review gives the reader a ride through strikingly diverse achievements obtained with TMV-based particles, compares them to the progress with related viruses, and focuses on latest results revealing special advantages for enzyme-based biosensing formats, which might be of high interest for diagnostics employing 'systems-on-a-chip'.

## Introduction

In the early years of virology, viruses were primarily regarded as small infective agents sometimes causing fatal diseases. Today, viruses and virus-like particles (VLPs) are seen in a different light by a steadily growing scientific community: A broad scope of interest was shifted towards the application of spherical and elongated viruses in nanotechnology [1–5]. As several viral particle types are supramolecular complexes of well-defined shapes and dimensions, and with numerous specifically addressable coupling sites exposed on their outer and sometimes inner surfaces, they offer intriguing possibilities for use as nanocontainers for the encapsidation or fabrication of compounds, or as nanofiber scaffolds for the immobilization and presentation of functional units. Most of the novel approaches make use of plant or bacterial viruses non-pathogenic for warm-blooded animals, or of non-infectious virus shells which lack part or all of the viral genome. Several viruses may be genetically engineered to increase the number of selectively addressable binding sites, or to alter specific properties of the template [6–7]. Principal players in virus-based nanotechnology are *cowpea mosaic virus* (CPMV), *cowpea chlorotic mottle virus* (CCMV), *potato virus X* (PVX), the bacteriophages MS2 and M13 and *tobacco mosaic virus* (TMV; for a list of abbreviations, see Table 1) [1,4,6,8]. The application possibilities for viruses are as versatile as the composition of the interdisciplinary teams working on them. In the following, we report on trends in the gradually changing field of research on virus and virus-like particles, with special focus on the well-known TMV nanosticks and their use as nanostructured scaffolds for the efficient and advantageous display of biomolecules, namely of active enzymes.

**Table 1:** Abbreviations of biological/biochemical building blocks at a glance.

abbreviation	description

4CL2	4-coumarate:CoA-ligase 2
ABTS	2,2′-azino-bis[3-ethylbenzothiazoline-6-sulphonic acid] (chromogenic HRP substrate)
A-protein	small oligomers of tobamoviral CP subunits
'Biotin-'	'maleimide-PEG_11_-biotin linker-' (in compound forms: biotin linker-equipped unit)
CALB	*Pseudozyma (Candida) antarctica* lipase B
CCMV	*Cowpea chlorotic mottle virus*
CPMV	*Cowpea mosaic virus*
CP	coat protein
CP_Cys_/Bio	coat protein of TMV_Cys_, equipped with biotin linker
disk	ring-shaped aggregate of 34 CP subunits (two layers of 17 CP molecules each)
FMDV	*Foot-and-mouth-disease virus*
GOx	glucose oxidase
GPx	glutathione peroxidase
GSH	glutathione
HRP	horseradish peroxidase
ICTV	International Committee on Taxonomy of Viruses
OAs	origin of assembly (RNA sequence nucleating tobamovirus self-assembly)
PDMS	poly(dimethylsiloxane)
PVA	*Potato virus A*
PVX	*Potato virus X*
[SA]	streptavidin
[SA]-GOx	streptavidin-conjugated GOx
[SA]-HRP	streptavidin-conjugated HRP
Sec	selenocysteine (cysteine analogue with selenol in place of thiol, in selenoenzymes)
STS	stilbene synthase
TLP	TMV-like particle
TMV	*Tobacco mosaic virus* (nanotubular, 'rod-shaped' nucleoprotein particle)
TMV_Cys_	TMV particles containing S3C-mutant CPs exposing a thiol on every CP_Cys_
TMV_Cys_/Bio	TMV_Cys_ equipped with [maleimide-coupled]-PEG_11_-biotin linkers
tobamovirus	plant virus genus (taxonomic group, type member: tobacco mosaic virus)
VLP	virus-like particle
ZYMV	*Zucchini yellow mosaic virus*

## Review

### From virus discovery to current research trends

Around the turn of the 19th century, a new type of infectious agent, the 'virus', was discovered [9–10]. During the following decades it was proven that all viruses consist of genetic DNA or RNA material surrounded by a protein coat (capsid), and optionally a lipid envelope. Since virus genomes do not encode all biochemical mechanisms necessary for their own replication and spread, they exploit the genetic repertoire of the infected host cells, which are re-programmed to produce many thousands of virus copies. Viruses have been discovered in virtually all types of organisms, with numerous well-characterized species adapted to animals, plants, fungi, or prokaryotic bacteria or archaea. As they may have an enormous impact on the physiological status and health of the host, viruses were originally defined and classified in regard to the diseases provoked, and to their minuscule size due to which they passed sterilization filters. The second half of the 20th century is often referred to as "the golden age of virology": During these decades, several Nobel Prizes were awarded for the description of new viruses and important insights into the characteristics of virus–cell interactions [11]. A virus taxonomy with a constantly developing classification and nomenclature was introduced by the International Committee on Taxonomy of Viruses (ICTV) [12]. The most recent ICTV report from the end of 2011 [13] lists 87 families with 2,284 virus and viroid species and many yet unassigned viruses, which had increased to 3,186 species by end of 2015 [14]: The identification of further viruses is accelerating rapidly due to next generation sequencing techniques revealing a plentitude of so far unknown, often inconspicuous viruses in the environment. With the growing knowledge on virus diversity it becomes more and more obvious that numerous species are not associated with any disease, and others even exert positive effects on their hosts [10,15]. Viruses also contribute substantially to the horizontal exchange of genetic material not only within a single host species, but even between genomes of distinct organisms susceptible to the same virus [16]. Hence the discipline of virology experiences intense novel research activities towards a better understanding of the evolutionary roles and individual capacities of distinct viruses, and the mutual interactions between virus populations and communities ('viromes'), hosts and their environments. Concurrently, remarkable progress has been made in the investigation of natural multifunctional complexes, and the development of 'smart' hybrid structures with elaborate functionalities exploiting biologically optimized principles. As these are sought-after especially for novel miniaturized devices and hierarchically organized materials, a second strikingly expanding field in contemporary virus research addresses both organization and re-design of viral nanoparticles. As introduced above, these are attracting special attention as multivalent shells enabling an encapsidation, presentation and delivery of functional molecules in different setups, and as building blocks of nanoarchitectures performing complex tasks up to biochemical conversion. Most suited in this context are robust, self-assembling viruses non-pathogenic for mammals and their bacterial flora such as TMV.

### Tobacco mosaic virus

#### Historic cornerstones

**Figure 1:**
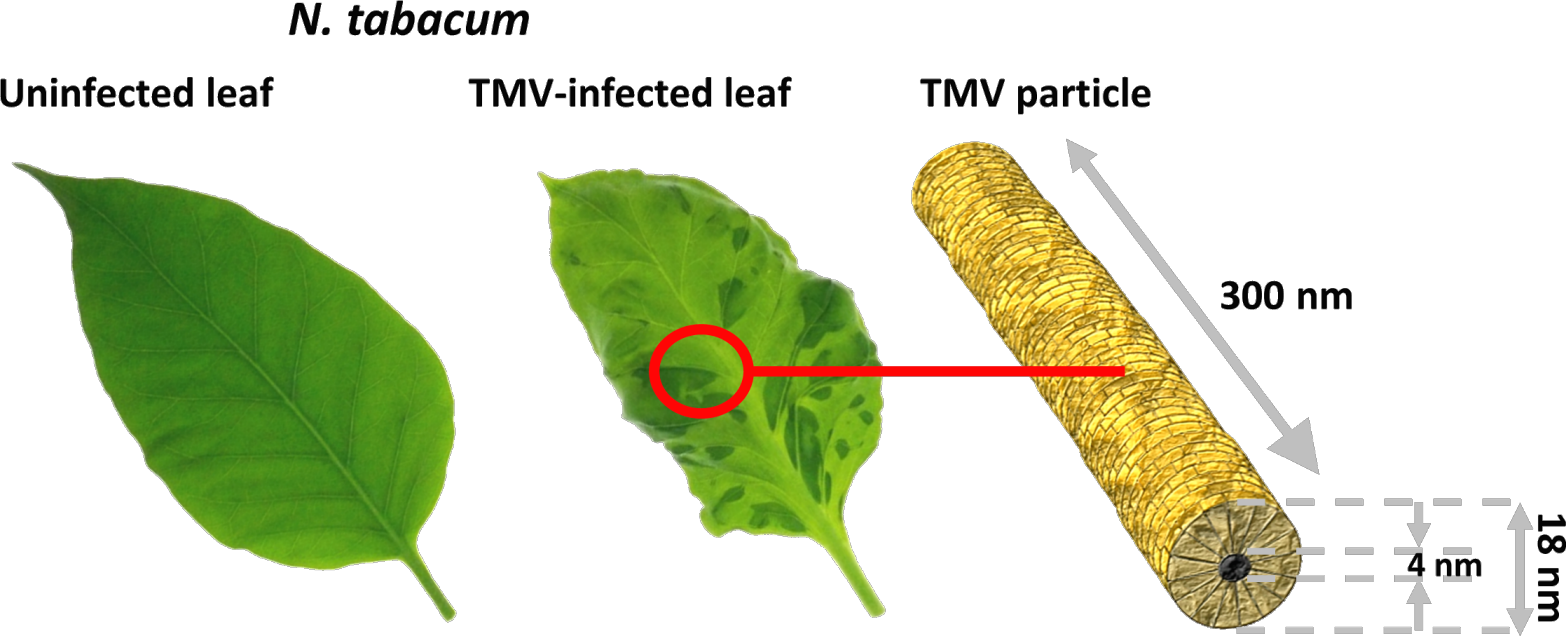
Tobacco mosaic virus infection. Left: Leaf of a healthy tobacco plant (*Nicotiana tabacum* 'Samsun' nn). Center: Leaf of a TMV-infected plant showing the typical TMV-associated mosaic (light and dark green mottling on the leaf blade). Right: Organization and dimensions of a TMV ribonucleoprotein particle, with the RNA not depicted as it is completely enclosed in the CP helix (golden).

The first plant virus ever described was the *Tobacco mosaic virus* (TMV; genus: *Tobamovirus*, family Virgaviridae [17]). In 1886, Adolf Mayer reported on a disease that had occurred for many years in tobacco cultivated in Holland, which was manifested by stunting and leaf symptoms including brittleness and a mosaic-like color variegation of light and dark green (Figure 1). Therefore he named the syndrome “tobacco mosaic” [18]. A few years later, Dimitri Ivanowski revealed that the extracts of mosaic-affected leaves remained infective after passage through bacteria-excluding filters [19]. Martinus Beijerinck, while repeating Ivanowski´s experiments, recognized that he was dealing with a pathogen of a novel type, much smaller than a bacterium, which he called “virus” in 1898 to indicate its non-bacterial nature [20–22].

From 1935 on, Wendell Stanley examined the tobacco and a similar tomato disease and attributed them to an infectious crystallizable protein [23–24]. Soon thereafter, Bawden and co-workers detected a nucleic acid fraction of about 5% in the fibrous crystals [25–26]. These were shown by Helmut Ruska and colleagues, who pioneered electron microscopy in Berlin at that time, to most likely consist of rods about 300 nm in length and 15 nm in diameter [27]. In the 1950s, cutting-edge X-ray diffraction studies along with intense scientific interactions of James Watson, Rosalind Franklin and Donald Caspar revealed the first major clues of the helical organization of the TMV nucleoprotein particles (for a thorough historic overview see [28]). They localized the viral genomic RNA strand wrapped in a helix of more than 2000 identical coat protein (CP) subunits, enclosing a hollow longitudinal channel of 4 nm diameter (see below, and Figure 1). The assembly mechanism of these nanotubes was a subject of intense research up to the late 1970s, with still some debate on details and putative different modes going on (see [29–32] for original research or for extensive reviews [33–36]). Concomitantly, infectivity and genetic organization of the viral RNA as well as the CP amino acid sequence were determined as reviewed in detail [37], with the complete RNA sequence published in 1982 [38]. Summing up, TMV was the first plant virus of which a highly resolved particle structure including the amino acid sequence of its protein, and all major gene functions had been unraveled. Furthermore, it was the first of all viruses with both assembly and disassembly shown to take place in vivo and, under suitable conditions, also in vitro [34,37,39–41]. TMV thus played a key role in the origin of the virology discipline [21], and in the advancement of bioanalytical technologies, with an exciting body of literature elaborating specific aspects [28,42–43].

#### Particle structure and assembly

**Figure 2:**
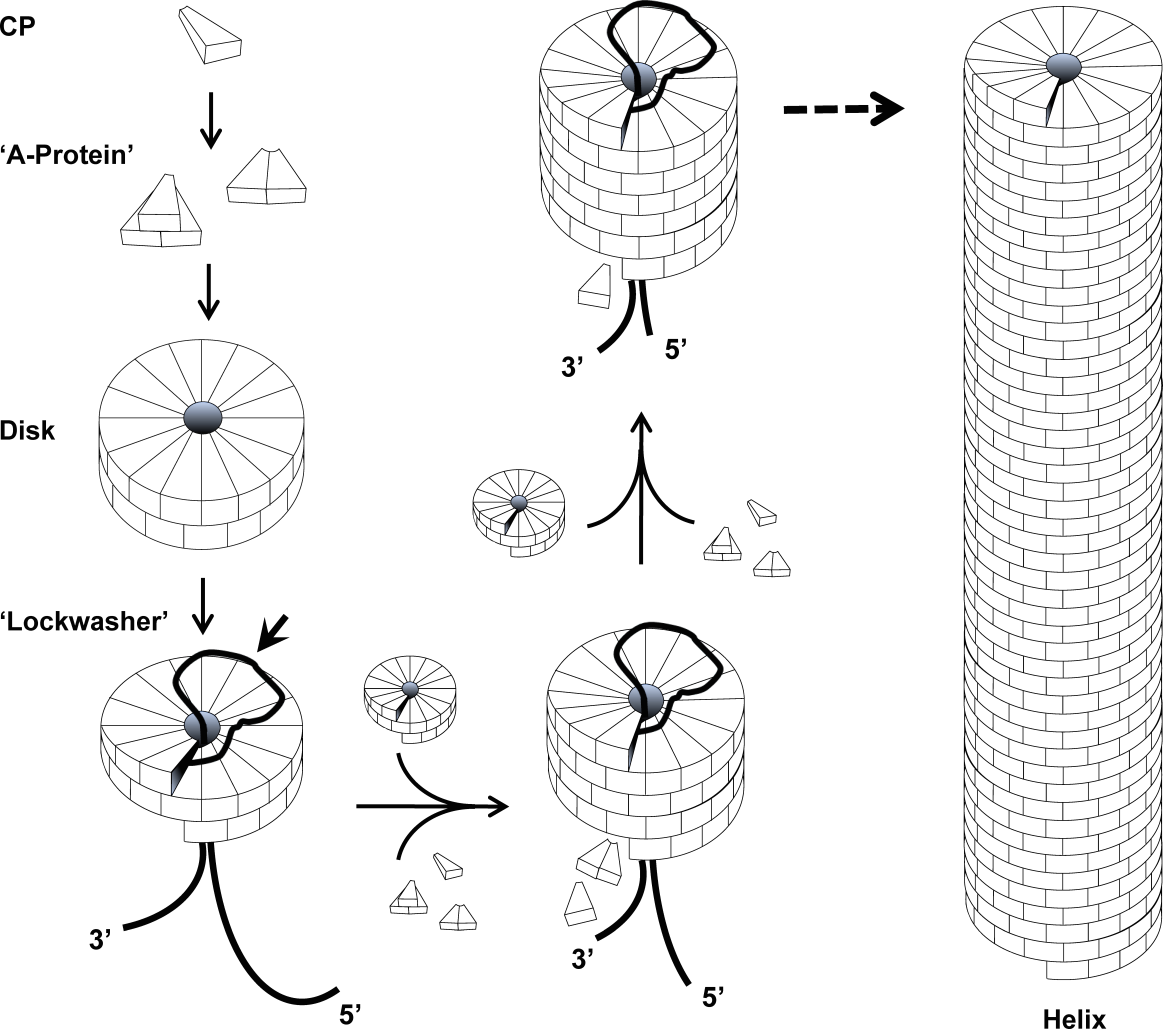
Self-assembly process of TMV. Model for the bidirectional self-assembly of nanotubular TMV particles, based on the formation and consumption of different CP oligomers, i.e. "A-protein" and disks, in vitro. RNA is shown as a black line. Assembly of TMV starts with insertion of the RNA *origin of assembly* (OAs)-loop into the central hole of a protein disk, resulting in its conformational change into a helical “lockwasher” and the formation of an RNA “traveling loop” (indicated with arrow) and integration of the adjacent RNA portion between the CP layers. Fast tube elongation towards the 5’-tail of RNA is achieved by the serial addition of protein disks, while slow 3'-elongation occurs through use of “A-protein”. Modified from [35].

TMV is a supramolecular ribonucleoprotein tube of ca. 2130 identical CP subunits, organized as a continuous right-handed helix enclosing a single-stranded viral RNA of 6395 nucleotides, which is helically integrated between the proteins and thus completely protected [44–46]. Each CP subunit consists of 158 amino acids. Numerous studies have investigated the self-assembly of TMV in vitro and provided many pieces of the puzzle, with most of them evidencing a bidirectional tube growth starting at an origin of assembly (OAs) site in the 3'-portion of the viral RNA [33,47], as illustrated in Figure 2. Depending primarily on pH, ionic strength, and total protein concentration, CPs may form small oligomers collectively known as "A-protein". This aggregation of TMV protein in the absence of RNA is mainly driven by hydrophobic interactions. CPs further assemble into higher-order ring-shaped aggregates of 34 subunits, each containing two layers of 17 molecules. These double-layered “disks” are essential for the nucleation of TMV particle assembly in vitro*,* as they may undergo a conformational change into a short helical “lockwasher” [33,48]. Most likely, the interaction of a disk with a specific RNA stem-loop of the OAs [49–52] triggers its transformation into the helical state, thereby threading the 5’-portion of the RNA into the nascent protein helix. It becomes incorporated between the CP layers and will form a “traveling loop” accessible on the “top” end of the particle [35]. Starting from this nucleation complex, a fast cooperative growth towards the 5’-tail occurs by serial addition of further “disks” and concomitant packaging of the 5'-segment of the RNA, which is drawn “up” through the central channel. Simultaneously, the 3'-portion of the RNA is encapsidated, but in a slower manner by the incorporation of small CP aggregates or “A-protein”. Several comprehensive reviews provide the details [33,35–36,47,50].

The complete final particle is 300 nm long, with an outer diameter of 18 nm and a central channel of 4 nm diameter [53]. With the aid of modified RNA molecules, TMV-like particles (TLPs) of altered length, or non-linear more complex structures up to branched architectures may be generated [54–59]. Furthermore, it is possible to immobilize one end of the RNA template prior to its encapsidation by CP, resulting in TLP growth bottom-up at sites of interest [54,57,60]. Finally, different TMV CP variants (mixed or arranged in domains) can be combined in single particles, which then may expose distinct chemically addressable surface groups [61–62]. This tunability in shape and biochemical properties makes TMV derivatives particularly versatile biotemplates for the fabrication of hybrid nanostructures and the high-density presentation of functional compounds.

### New applications of viruses in nanotechnology

#### Virus-based nanotechnology

**Figure 3:**
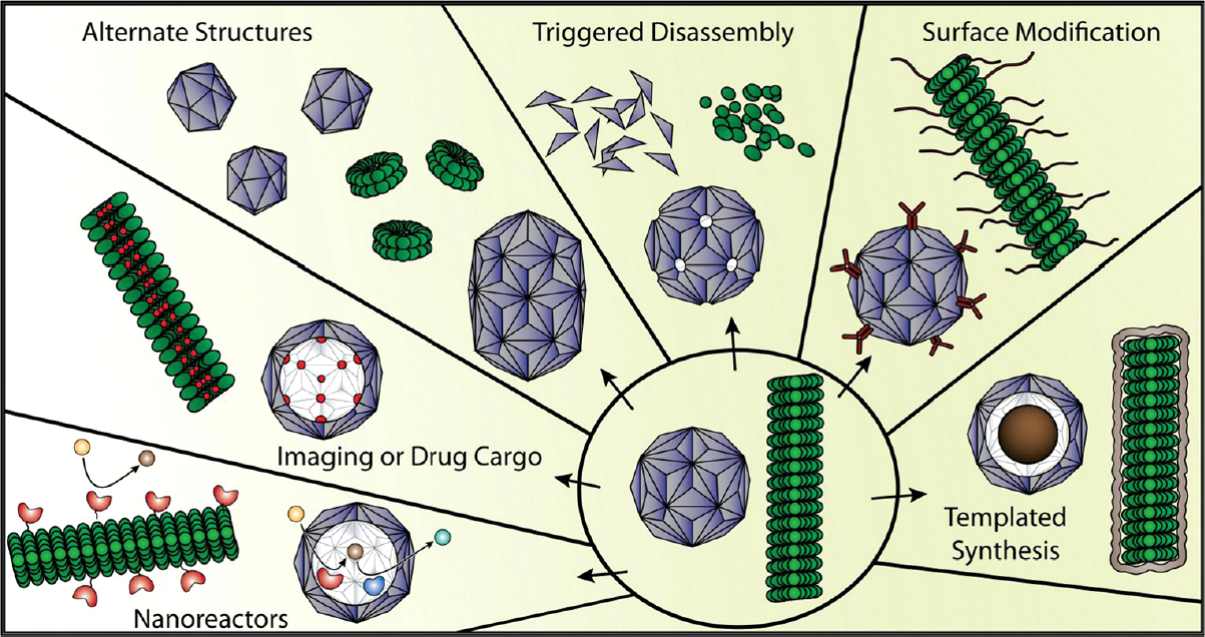
Fabrication of distinct types of viral capsids engineered for various applications. Virus particles of both spherical and helical shapes are accessible to a wide range of modifications, making them versatile tools e.g. for chemical synthesis, medical imaging or drug delivery applications, and biotemplates of variable shapes to serve as nanocarriers for functional units and building blocks of extended hybrid materials. Reproduced with permission from [6], copyright 2014 Springer.

In addition to virological research in the original sense, investigating, e.g., virus structure, replication, spread, or interactions with host cells and tissues, viruses and VLPs have been regarded as a new class of biomaterials with an immense potential for applications as templates, building blocks and “smart” tools in chemical synthesis, medicine and nanotechnology since the turn of the century [1–4,63–64]. Several virus types exhibit high stability and precise 3D structures and dimensions with well-defined interior and exterior surfaces. Some of them are accessible to controlled modifications and production in large quantities from renewable resources. These are being tested extensively as scaffolding biotemplates in various applications, ranging from biosensing to tumor therapeutics. Hence, in the recent past, the number of reports on the successful construction of advantageous virus-derived hybrid materials and devices has been growing rapidly and reviewed extensively [5,7–8,65–70]. Most viruses utilized as biological building blocks are plant or bacterial viruses of either spherical (quasi-icosahedral) or fiber/rod shape (helical symmetry), with the application perspectives differing in certain aspects for these two types of backbones (as illustrated in Figure 3 and some review articles [6,71–72]). Although they have in common that they are non-pathogenic for humans and animals, spherical plant viral or bacteriophage particles serve predominantly as nanocontainers for cargo or reaction vessels, and as presentation systems for bio-effective molecules such as antigens, whereas rod and filamentous backbones excel in the particle structures available, and the dense exposure of docking and chemically reactive sites attainable on their outer coat.

The functional molecules immobilized on, or encapsulated in viral capsid comprise, amongst many others, fluorescent dyes for imaging purposes either tracing the virus itself, or targeting specific cells or organs [73–76], antigens for the development of vaccines as reviewed in great detail [77–79], drugs or imaging reagents for increasingly elaborated biomedical applications [80–82] with numerous further examples described in overview articles [70,72,83–86], antibodies as virus-based tracers [65], immunoadsorbents [87] or molecular stickers [88], to name but a few examples. Much effort is also directed towards the fabrication of nanostructured hybrid materials with improved or novel chemical and physical properties, based on the combination of a multitude of synthetic and inorganic compounds with virus assemblies [5,7,68–69,89–90].

#### Application of TMV-/tobamovirus-based structures

**Figure 4:**
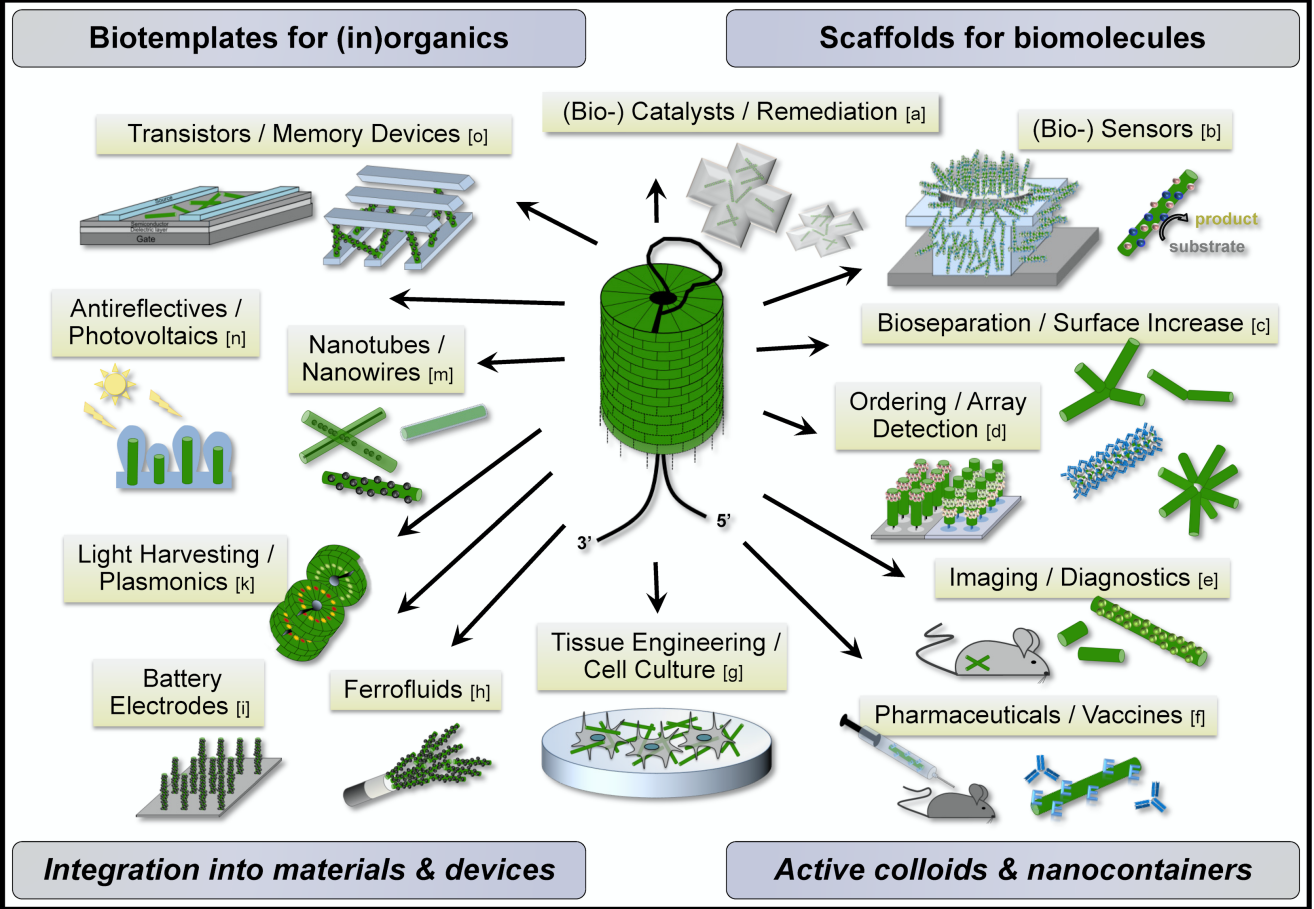
TMV and related tobamoviruses: versatile templates for the construction of biohybrid nanoobjects and nanostructured materials for a wide range of applications exemplified schematically. Among numerous reports on respective approaches, the following references may illustrate selected specific achievements and strategies: [a] = [113,133]; [b] = [132,134]; [c] = [57–58,87]; [d] = [54,61,135–137]; [e] = [116–118,121,124]; [f] = [125–127,129,138]; [g] = [122–123,139–140]; [h] = [59]; [i] = [107,141]; [k] = [119–120]; [m] = [93–96,98–99,102,105,111]; [n] = [109]; [o] = [103,110,112].

In these recent developments, TMV with its rigid, self-assembling nucleoprotein capsid continues to play a leading role among the most promising viral backbones [5,7,91–92]. TMV and closely related tobamoviruses have been applied as versatile templates for the metallization or mineralization of their inner or outer surfaces, respectively, yielding nanosized components for, e.g., electronic devices, batteries, photoelectrochemical cells or environmental remediation [63,93–113], as scaffolds for the display of reporter dyes, nanoparticles or contrast agents such as gadolinium complexes, for light harvesting, energy conversion, plasmonics or magnetic resonance imaging [82,92,100,114–121], and as carrier rods for effector peptides for distinct purposes from affinity binding, intravital targeting up to cell-culture supports [105,111,117,122–124], or as antigens for vaccination [125–129]. Finally, the exposure of enzymes or biocatalytically active domains on the TMV surface is an area of intense research, since the viral nanoparticles are expected to offer specific advantages over conventional immobilization matrices [130–131]. These approaches, however, bear special challenges as genetic fusions to the TMV CP are tolerated only to a limited extent [47,88,131]. That is why up until now, the number of respective studies is low, though particularly promising [132–133], as described in the following. The manifold functionalization and assembly procedures applied to tobamoviral scaffolds have yielded a multiplicity of biohybrid nanostructures for strikingly different uses, as illustrated by Figure 4, which also provides corresponding references.

#### Enzymes presented by viral carrier templates: promises, limits and prospects

Enzymes are extensively used in the industrial fabrication of chemicals and pharmaceuticals as well as in analytical routines. There is a considerable interest to steadily increase productivity by optimizing process conditions, engineering the operational performance of the enzymes, and improving immobilization techniques towards a better handling of fully active enzyme preparations. A broad range of different immobilization methods is applied, ranging from protein adsorption, covalent coupling, entrapment and encapsulation to intermolecular cross-linking with filler components, specific high-affinity docking and combinations thereof [142–144]. Enzymes installed on solid supports often exhibit enhanced stability, in comparison to their soluble counterparts [145–146]. One important additional aspect is their simple separation from the reaction solution and the resulting reusability [147]. On the other hand, immobilization may lead to reduction or even complete loss of enzymatic activity [148], as a result of conformational changes or blockage of the active sites due to interactions with the coupling matrix [149–151]. Therefore, advantageous strategies to immobilize and present enzymes in a predictable and sterically favorable orientation at high surface densities are desired. Virus-derived templates appear to be among the best-suited carrier backbones in this context, since they provide all prerequisites to achieve tight positional control of the bioactive units [130], and may ensure optimal steric accessibility of the catalytic centers if protruding into the reaction liquid. This would be realized best with rigid rod-like carrier objects such as TMV and TLPs deduced thereof.

Up to now, however, the application of viral scaffolds for the presentation of enzymes on their outer surfaces has rarely been investigated, though with promising prospects in most of the few cases published [88,132,152–155]. By contrast, enzyme encapsulation inside viral cavities is a matter of much broader research [72,156–157], in analogy to synthetic polymersomes that are tested extensively for uses as nanoreactors [158–161].

This article delivers insight into recent achievements on TMV-derived carrier rods equipped with biological enzymes. First, though, a glance at the other successfully established hybrid complexes of elongated plant viruses fashioned with active enzymes introduces typical obstacles. As explained thoroughly by Cardinale and co-workers [130] and by Pille et al. [88], a major problem lies in the low tolerance of most such viruses for accepting major alterations of their CPs, including extensions and charge modifications. Viral filaments containing a single, densely packed protein species are especially prone to structural disturbances, which may abolish assembly of the viral particles due to steric constraints. This often promotes back mutations and thus loss of engineered CP domains. Furthermore, changes of the viral surface may also provoke defense reactions up to tissue necrosis aborting viral multiplication, which has been analyzed in detail especially for TMV [47]. Consequently, direct genetic fusion of enzyme sequences to the ends, or into exposed loops, of viral CPs frequently failed, and also short peptide tags enabling bioaffinity linkage were inserted only occasionally [87,94,134,162]. Heterologously expressed TMV CP species offer more degrees of freedom for modifications. However, CP from *E. coli* does not organize into TLPs with RNA efficiently since it lacks an N-terminal post-translational acetylation; and up to now the yield of TLPs from eukaryotic yeast cells is relatively low [55]. Hence ectopically produced TMV templates are not yet accessible. Finally, an attachment of functional molecules to virus surfaces equipped too densely with anchoring tags may also be inefficient.

For these reasons, previous work on elongated viral enzyme carriers succeeded only if elaborate conjugation strategies were applied, addressing a subset of the viral CPs or introducing connector molecules. Direct genetic extension of about 60% of the roughly 1,300 CP subunits of PVX (genus: *Potexvirus*) with *Pseudozyma (Candida) antarctica* lipase B (CALB) was achieved by help of the ribosome “skip” mechanism ([154] and T. Michon, personal communication), which is attained by foot-and-mouth-disease virus (FMDV) 2a peptide-like junctions [73,163]. Although the enzyme CALB underwent a 45-fold decrease in activity, it functioned stably on the plant viral backbone. The use of antibodies as “molecular stickers” and thus spacers was developed into a partially universal coupling concept to viral templates [88]: It allows for the stable binding of functional proteins fused with the Z33 peptide (derived from staphylococcal protein A [164]) to the Fc portions of immunoglobulins IgG_1_, of which high-affinity species directed against various virus types are available. By these means, a fully active 4-coumarate:CoA-ligase 2 (4CL2; N-terminally fused to Z33) was installed on a distinct filamentous plant virus, *zucchini yellow mosaic virus* (ZYMV, genus: *Potyvirus*), with almost 90% surface coverage [88]. This work has recently been extended to a related potyvirus, *potato virus A* (PVA), which could be fashioned with similar surface density not only with 4CL2, but also with a two-enzyme mix of 4CL2 and stilbene synthase (STS) expressed in *E. coli,* or with a fusion protein of both partner enzymes [155]. The blended enzymes were able to collaborate in a cascade reaction, converting *p*-coumaric acid and further precursors into resveratrol. This polyphenolic compound is well-known to wine connoisseurs, since it is found predominantly in red grapes and wine and has been extensively discussed to promote health. Although, to date, the fusion protein has not exhibited detectable activity on PVA, and the resveratrol yield from the 4CL2/STS-PVA assemblies was relatively low, the functionality of the enzyme cascade was clearly demonstrated [155]. Further optimization and transfer to related enzymes might therefore result in biological production platforms for resveratrol or further “functional food” ingredients, employing potato or other plant-virus derived biotemplates as an immobilization matrix. The strategies shown to work for the use of enzymes on TMV carrier particles are presented in more detail in the next section.

### TMV as carrier for biological enzymes

#### Linker-enabled bio-affinity coupling of enzymes to TMV

**Figure 5:**
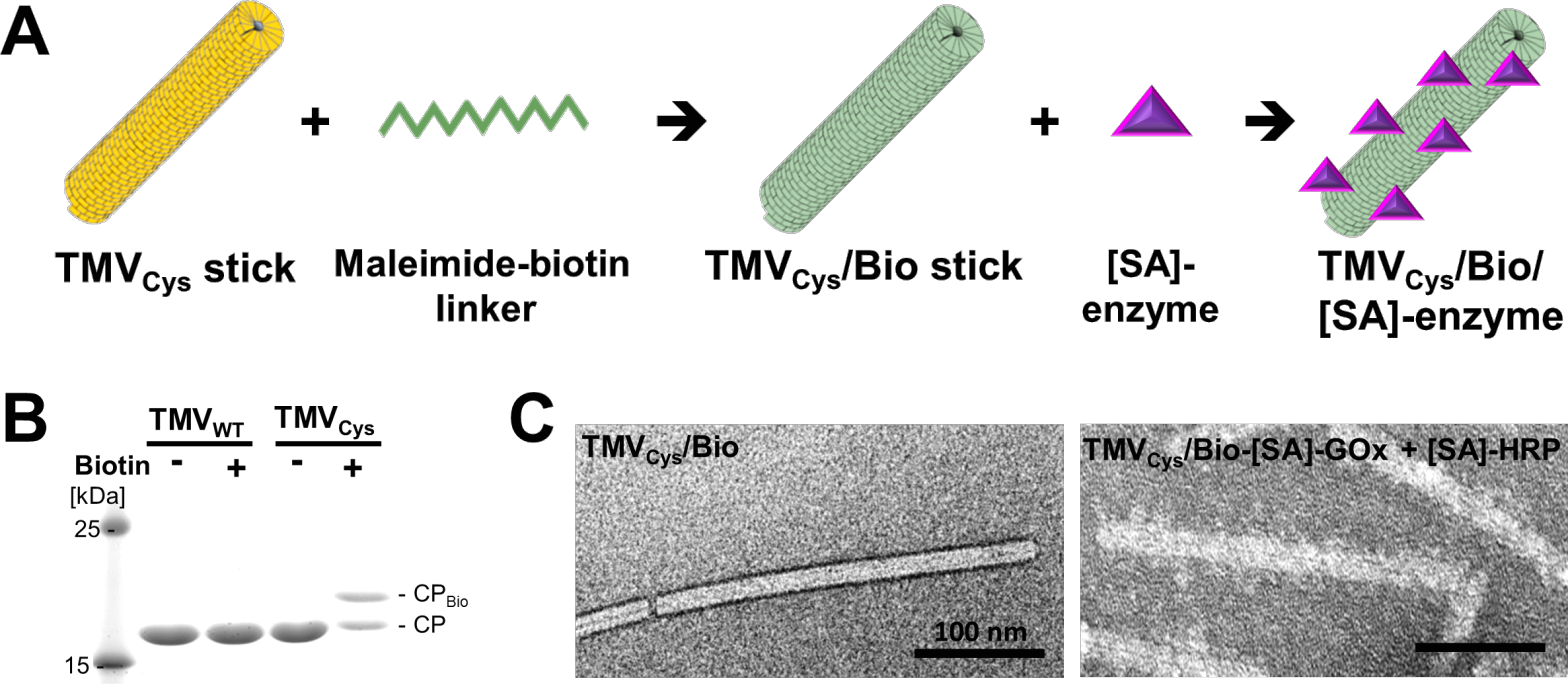
TMV equipment with biotin linkers and [SA]-enzymes. A: Scheme of TMV functionalization. A coupling-competent TMV variant (TMV_Cys_, yellow) was coated with bifunctional maleimide-PEG_11_-biotin linkers resulting in TMV_Cys_/Bio (green), followed by affinity binding of [SA]-enzymes (purple). B: Selective linker coupling to CP_Cys_ (17.6 kDa) was possible with ca. 50% efficiency, as shown by specific gel shift of the CP_Cys_ band (CP_Bio_). Molecular weight marker bands as indicated (15% SDS-PAGE stained with Coomassie Brilliant Blue R250). C: Transmission electron microscopy of TMV_Cys_/Bio particles before and after binding of [SA]-enzymes (mix of [SA]-GOx:[SA]-HRP 14:1). The enzymes form a fluffy seam with protrusions, distributed over the whole particle length. Samples were adsorbed on Formvar^®^-coated carbon-sputtered copper grids and negatively stained with 2% uranyl acetate; bars: 100 nm. Images show data obtained according to reference [132].

**Figure 6:**
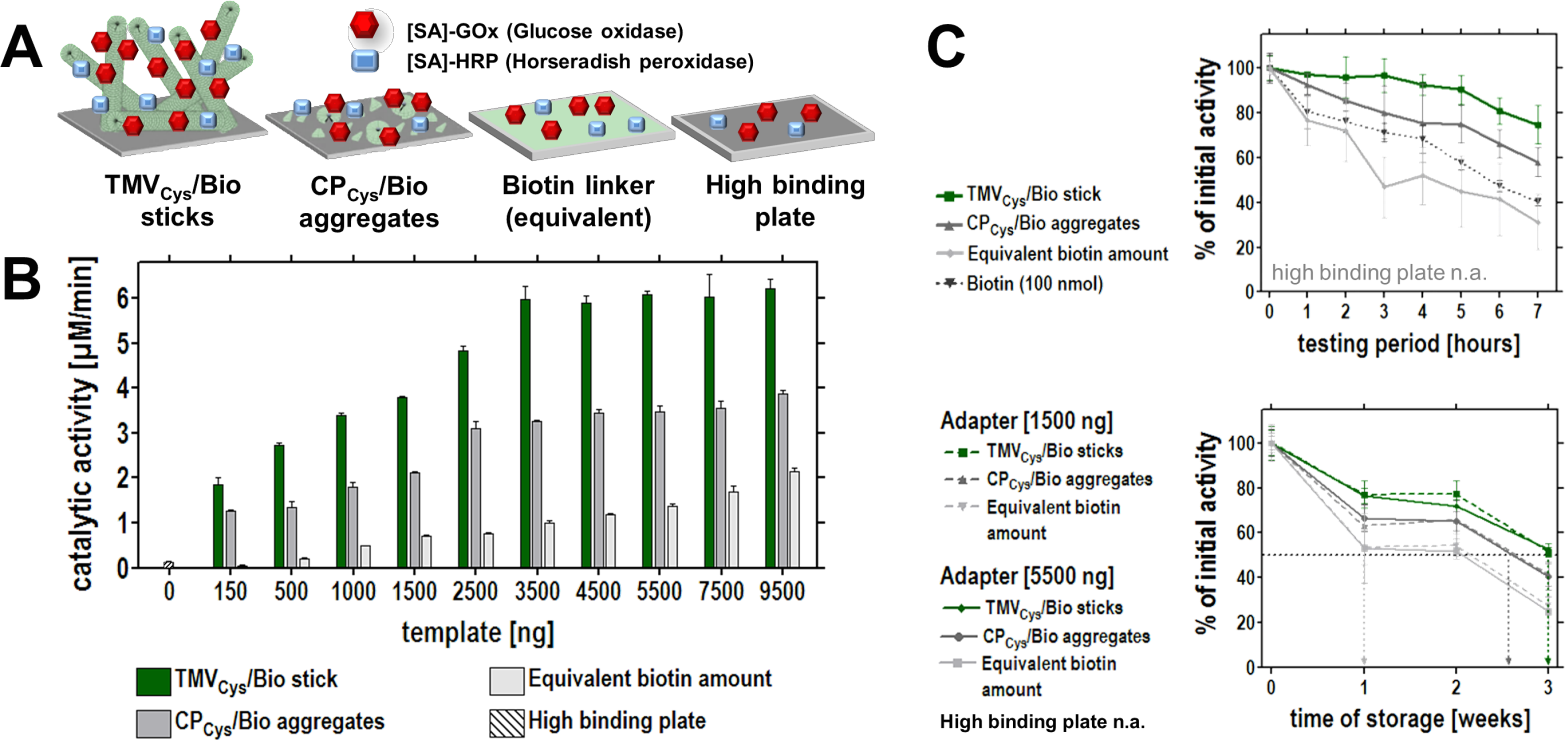
Influence of TMV adapter scaffolds on enzyme-based glucose detection layouts. A: Schematic drawing of different detection layouts using TMV-derived or linker adapters for the immobilization of enzymes on solid supports, or adsorptively immobilized enzymes on untreated surfaces (layouts with adapters: with TMV_Cys_/Bio rods, CP_Cys_/Bio aggregates, or biotin linkers; without adapters: plain surface). B: Catalytic activities achieved with these layouts using different adapter concentrations, applying the same [SA]-GOx/[SA]-HRP input for colorimetric glucose detection *via* formation of ABTS* radicals (absorption maximum at λ = 405 nm). TMV adapter templates support the immobilization of substantially increased enzyme activities. C: TMV adapters exert enzyme-stabilizing effects, increasing both the reusability of the immobilized enzyme upon hourly repeated uses (top), and the storage stability over a testing period of three weeks. Initial turnover rates were set to 100 % and the percentage of remaining activities calculated. Degree of reusability: TMV_Cys_/Bio nanorods > CP_Cys_/Bio aggregates > biotin linker > substrate lacking any adapter molecule. For details, see text and reference [132] for details. B/C: Reproduced according to the Creative Commons Attribution 4.0 International Public License from [132].

A recent study sought to find out if the increasing use of rigid TMV templates might also be extended to the immobilization of enzymes [132], as demonstrated for flexuous PVX and ZYMV particles. Since diagnostic assays could profit particularly from novel nanosculptured high-density presentation platforms, a glucose-sensing enzyme combination of glucose oxidase (GOx) and signal-generating horseradish peroxidase (HRP) was applied (see below in Figure 5 and Figure 6). Due to the promising and in part unexpected findings, some details of the approach are reported here exemplarily.

To circumvent the need for extensive changes to the natural TMV structure, the enzymes were immobilized on the surface of moderately engineered TMV sticks by a two-step procedure [132]. This combined the covalent coupling of biotinylated linkers to a tailored virus variant, and subsequent bioaffinity binding of commercially available streptavidin [SA]-enzyme conjugates (Figure 5A). A genetically modified TMV (TMV_Cys_) with a cysteine residue (S3C) surface-exposed nearby every CP N-terminus [61] served as a carrier rod with more than 2,000 addressable thiols. These were equipped with maleimide-reactive bifunctional linkers (maleimide-PEG_11_-biotin; see [165] for details on bioconjugation techniques), resulting in biotinylated TMV_Cys_/Bio. The PEG_11_ linker spacer provided increased degrees of freedom for a dense decoration with [SA]-enzymes, and a spatial decoupling of the enzymes and the TMV scaffold.

About 50% of the CP_Cys_ subunits could be decorated with biotin linkers, and virtually all thereof with enzymes (Figure 5B,C), resulting in complete coverage of the TMV sticks by more than a single layer: The volume of all immobilized enzyme molecules surpassed what was available directly around the TMV surface. This indicated that the flexible spacer arms of the linkers enabled a staggered high-density arrangement of the enzyme conjugates.

The two-enzyme cascade system of GOx and HRP enabled a colorimetric detection of catalytic activity and the quantification of glucose [152,166–167]. Specifically, GOx catalyzes glucose oxidation to D-glucono-1,5-lactone, thereby producing hydrogen peroxide [168]. This is a substrate for HRP, which reduces it to water, and, as a side reaction, can convert a chromogenic substrate (e.g., tetramethylbenzidine TMB, diaminobenzidine DAB, or 2,2′-azinobis[3-ethylbenzothiazolinesulfonic acid] ABTS) into a colored product allowing for spectrophotometric detection [169]. Partitioning experiments tracing the fractionation of GOx/HRP activities after defined [SA]-enzyme input revealed that enzyme binding to the TMV_Cys_/Bio sticks did not affect their performance [132].

Taken together, the technically simple linker-mediated affinity binding of functional proteins turned out to be a straightforward approach for installing sensor enzymes on rigid TMV nanorods: the two-step method achieved maximum surface occupation with full enzymatic activity retained on the viral backbones.

#### TMV adapter scaffolds conducive to biosensing layouts

A main goal of the case study on TMV enzyme carriers was to find out if biodetection setups would profit from an integration of these multivalent nanobiotemplates, to increase the surface density and/or steric accessibility of enzymatic sensor components arranged thereon. Hence, the effects of two different TMV-derived adapter scaffolds conveying specific binding of [SA]-enzymes to conventional high-binding microtiter plates were analyzed in colorimetric assays, detecting the generation of ABTS* radicals (Figure 6). Equal enzyme input amounts were applied into plate wells coated with (i) either fully assembled biotinylated TMV_Cys_/Bio adapter sticks, or (ii) released lower-order CP_Cys_/Bio aggregates, (iii) or stoichiometric amounts of maleimide-PEG_11_-biotin linkers, respectively, or into (iv) uncoated plates resulting in mere enzyme adsorption (Figure 6A).

Assays in the presence of TMV adapters were substantially superior to the other layouts: In parallel experiments with variable adapter concentration, up to 45-fold higher substrate turnover rates were achieved with rod templates, and up to 25-fold higher with CP aggregates, whereas corresponding amounts of directly applied linkers resulted in a maximum 7-fold increase compared to the values obtained with directly adsorbed enzymes. Hence, the virus templates allowed a particularly efficient immobilization of active enzymes and thus strongly enhanced analyte turnover.

Beyond that, complete TMV rods exerted additional unforeseen beneficial effects on the performance of the enzymes. Increased reusability, greater stability, higher regenerability and higher analysis rates; these are but a few of many benefits appropriate enzyme immobilization routes may bring about [145–147]. To gather information about putative advantageous or deteriorating effects of TMV carriers on the long-term operation of sensor enzymes, the detection layouts in the absence and presence of the different adapters were tested extensively for reusability and storage stability [132]. Upon repetitive hourly uses, sample wells equipped with TMV_Cys_/Bio adapter rods exhibited remarkable remaining activities of around 90% upon the sixth, and 75% upon the eighth use (Figure 6C, top). Through three weeks of wet storage with 14 uses or idle, 40 to 50% of the glucose sensing activity was still retained (Figure 6C, bottom and [132]). TMV CP aggregates were able to stabilize the GOx/HRP system to a considerably lesser extent, but significantly superior to plain biotin linkers, whereas directly attached enzymes had lost detectable activities already after the first testings. In conclusion, the surface of assembled TMV rods seems to act as an activity-preserving environment for sensor enzymes. In combination with the sterically controlled high-density exposure possible on the nanostructured TMV surface, TMV-like carrier architectures may constitute supremely efficient immobilization supports worth testing in further detection setups.

#### Making TMV an enzyme itself

**Figure 7:**
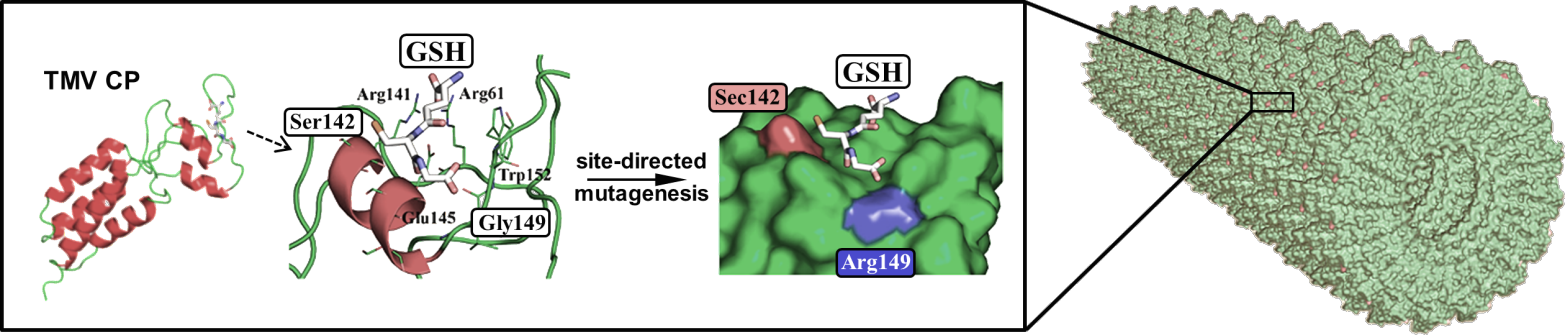
Transformation of TMV CP molecules into enzymatically active rod-like assemblies. By exchanging suitably exposed natural surface residues of a cysteine-free CP mutant for an Arg residue (interacting with reduced glutathione), and a Sec moiety introduced at a cysteine codon through an auxotrophic bacterial expression system (by replacing canonical cysteine), GPx activity could be conferred to the TMV CP building blocks. Under conditions favoring their self-assembly devoid of RNA, enzymatically active nanotubes with H_2_O_2_-reducing outer surfaces were generated. Adapted with permission from [133], copyright 2012 American Chemical Society.

The conformation of the densely packed CP subunits of TMV rod and disk assemblies results in a surface-relief with regularly ordered nanometric grooves and cavities (see [46] for the most recent high-resolution cryo-EM reconstruction of TMV at 3.35 Å resolution). These have inspired a striking piece of investigation, as one of the repetitively arranged depressions resembles the substrate-binding site of glutathione peroxidase (GPx, EC 1.11.1.9), a natural selenoenzyme: Computer-aided design identified adjacent, adequately positioned amino acids of the viral CP which offered the chance to be modified into a glutathione-attracting pocket containing a catalytic selenocysteine (Sec) moiety [133]. A corresponding project thus investigated if by means of respective mutations, an appropriate TMV CP variant could be transformed into an enzymatically active, assembly-competent building block for the fabrication of TMV-based artificial nanoenzymes mimicking GPx. This was realized with convincing results by the replacement of a suitably exposed serine by a cysteine residue, which then was equipped with its analogue Sec through heterologous expression in a bacterial system auxotrophic in cysteine (Figure 7). Not only did this protein organize itself into higher-order complexes under suitable conditions, after trapping reduced glutathione (GSH), the resulting disks or TMV-like nanotubes exhibited strong H_2_O_2_-reducing GPx activity comparable to that of natural GPx enzymes in the case of disk-like structures. The glutardialdehyde-stabilized complexes with multiple artificial biocatalytically active centers were shown to exert protective antioxidative effects on mitochondria, and might be of high value in biosensor setups for the enzymatic detection of reactive oxygen species [133]. Hence, the precise proteinaceous 3D structure of TMV may even be converted into novel types of designer rods with enzymatically active surfaces.

#### Perspectives on applicable TMV-assisted layouts

**Figure 8:**
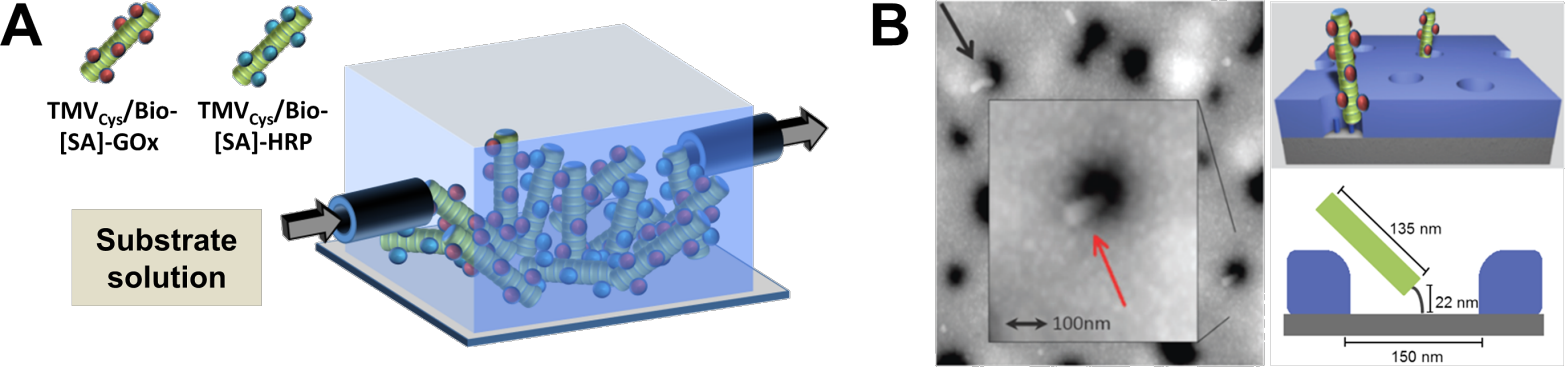
Prospects for novel layouts enabling lab-on-a-chip applications and improved orientation of enzymatically active TMV rods. A: Scheme of a microchannel of a glucose sensor chip equipped with a blend of two TMV enzyme nanocarrier species. By an appropriate mixture thereof, an optimally composed two-enzyme cascade of [SA]-GOx and [SA]-HRP can be established in a PDMS flow cell. B: TMV-like particles mounted in an upright position in a nanowell template fabricated as follows: A flat substrate was equipped with ssDNA and covered with a 60 nm thin, perforated metal-organic film produced by metal polymer blend lithography, followed by a metal-organic build-up reaction. OAs-containing RNA was subjected to hybridization with DNA on the bottom of the holes, then assembly-competent CP was added to yield TMV-like rods within the pores. The AFM Image (left) shows two TMV rods (black and red arrow) protruding from the 60 nm deep holes.

Among the many ideas about the technical uses of the rigid TMV nucleoprotein tubes, those which employ manageable amounts of viral templates and facile preparation strategies will have the best prospects for realization. Based on the promising findings on the performance of enzyme and antibody moieties installed [87,132,134] or engineered [133] at high surface densities on TMV nanocarriers, miniaturized sensor devices might be among the layouts worth extensive testing. Research on microfluidic lab-on-a-chip biodetection systems started in the end of the 20th century, and has been growing enormously since then [170–171], which parallels the investigation of viral biotemplates (see above). The main emerging application area is medical diagnostics, namely “point-of-care” on-site analytics circumventing the need for expensive instrumentation [172], with further uses becoming apparent in environmental, food, or intoxication diagnostics (for a recent detailed review, refer to [171]). Lab-on-a-chip solutions may integrate sample preparation and enrichment routines with multiple diagnostic tasks, requiring low total amounts of analytes as well as biorecognition elements if high local concentrations are achieved. In this regard, combinations of plant virus-based immobilization platforms with accordingly designed flow cells, as indicated schematically in Figure 8A, may be profitable and practicable.

Poly(dimethylsiloxane) (PDMS), a standard polymeric material used for the fabrication of microfluidic devices by master-replication techniques, was already shown to allow a site-specific bottom-up integration of TMV carrier sticks [60]. This was achieved via isothiocyanate- (ITC-) based coupling of single-stranded (ss) DNA anchors, subsequent trapping of the 3'-ends of assembly-directing RNAs with OAs, and their encapsidation by means of appropriate TMV CP preparations [54,60]. The resulting arrays of 3'-terminally fixed nanosticks are then accessible to biomolecule binding. Alternatively, pre-assembled TMV sticks loaded with functional molecules may be hybridized to ssDNA anchors after partial TMV disassembly, exposing a 5'-terminal stretch of their RNA, e.g., under mild alkaline conditions, as it has been demonstrated for TMV labeled with distinct dyes [135–136]. This allows for the installation of blends of distinct TMV-exposed effector molecules (as exemplified for [SA]-GOx/[SA]-HRP in Figure 8A), which is of special interest for the construction of enzyme cascade reactions, but also for multiplexing in advanced diagnostic approaches. Finally, adsorptive deposition of densely packed rod layers and three-dimensional assemblies was achieved for different TMV variants and solid supports, in some cases involving thiol bonding ([7,173] and references therein; [110,174–176]).

So far, however, it remains to be tested whether TMV particles immobilized via protruding RNA ends or through non-covalent attachment would retain their arrangement inside the flow channels of microfluidic devices in operation (for typical layouts and flow rates see [177]). To ensure robust implantation, full accessibility and constant spacing, ongoing work is attempting to fix TMV adapter sticks in upright positions. Initial results indicate that nanopatterned metal-organic layers might enable a fine-tuned adjustment of rod orientations in the desired dimensions (Figure 8B): Substrates coated with DNA anchors were equipped with a nano-perforated metal-organic layer via metal polymer blend lithography [178–179], and its topographical contrast amplified by a metal-organic build-up reaction. The resulting nanowell template was incubated with OAs-containing RNA, to allow its hybridization to ssDNA exposed on the bottom of the holes. After assembly-competent CP was applied, TMV-like rods were detected protruding in almost upright orientations (Figure 8B). Further improvements of the procedure might lead towards tight spatial control over the positions of the enzyme nanocarriers, which could be of high interest also for basic research on prerequisites for efficient enzymatic cooperation.

**Figure 9:**
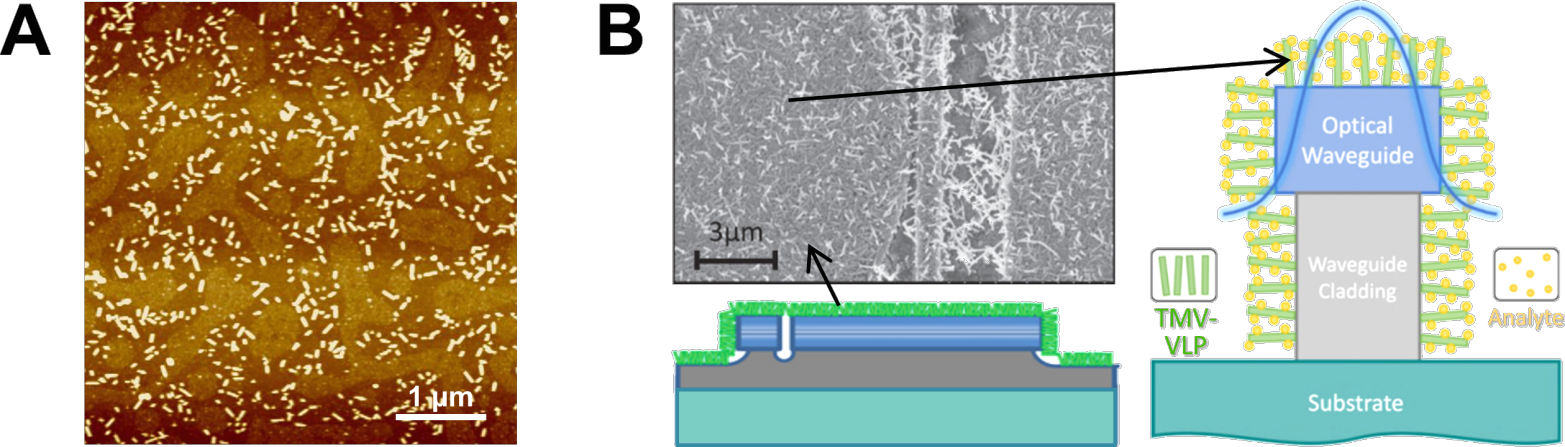
Arrays of TMV nanorods established by bottom-up or top-down approaches: Site-selectively arranged carriers for uses in biosensor devices. A: Spatially selective bottom-up growth of terminally immobilized TMV-like particles on aldehyde-modified areas of wafers, fashioned with assembly-directing RNA. After decoration with bioactive molecules, such arrays ensure stable interlinkage of technical surfaces and biological effector molecules. Adapted with permission from [54], copyright (2011) American Chemical Society. B: Engineered TMV-like particles coating the waveguide setup of an optical disk resonator serve as receptor layer for label-free biosensing. Analyte capture induces a shift in the resonant wavelength of the sensor cavity as a whole. Reproduced and adapted with permission from [134], copyright 2015 IOP Publishing. See text for additional information.

Fast, sensitive and cost-saving biosensors often employ label-free read-out, in which signal transduction occurs without a need of reporter molecules, e.g., by optical, electrochemical, mass- or surface plasmon resonance-sensitive techniques [171–172]. The development of site-selective and highly efficient methods for the application of TMV on inorganic or polymeric surfaces (Figure 9) has paved the way for the use of TMV derivatives as adapter scaffolds for sensor enzymes, or as receptor layers for bioaffinity-based recognition in advanced sensor configurations. Last year, a fundamental study evidenced that a capture of target molecules by engineered TMV-like particles, deposited on optical microdisc resonators, was readily detectable through changes in the effective refractive index of the waveguide setup and the concomitant shifts in the resonant wavelength of the sensor cavity, with signal amplification through label-free enzyme-linked immunosorbent assay (ELISA) [134]. The sensor system utilized RNA-free TMV-like rod assemblies fashioned with antigenic peptides as receptor layer, serving as haptens for the binding of specific antibodies. Its further development aims primarily at the construction of widely applicable biosensing devices recording biological antibody-target interactions, as they are used in numerous diagnostic routines. Label-free biodetection systems exploiting the beneficial effects of TMV adapter scaffolds carrying sensor enzymes are in the pipeline of current research efforts as well, and might promote novel diagnostic layouts for smaller, non-antigenic but bioconvertible analytes.

## Conclusion

TMV has accompanied human history for more than a century, threatening farmers and the tobacco industry as well as supporting and inspiring scientists. Once a key player in the origins of the virology discipline, enabling breakthrough findings on virus structure, properties and interplay with the host, concomitantly advancing the development of analytical techniques, the virus currently adapts novel roles: As a richly available, particularly robust biomolecular complex, TMV allows for extensive exploration studies on prospects and definite limitations of novel synthetic, nanostructured protein-containing material combinations. With respect to the general knowledge that a lot of frogs need to be kissed (or, originally spoken, thrown at a wall) before meeting a handsome prince [180], research on advantageous applications of TMV nanorods has yielded exceptionally promising perspectives in a fairly short period of time. These have been outlined throughout this review, against the background of typical problems encountered upon modifying viral constituents, and in relation to progress obtained with distinct elongated plant viral backbones.

It is only within the past few years that plant-made effector proteins, pharmaceuticals and virus-like particles especially for vaccination purposes are regarded economically valuable, attractive alternatives to conventionally produced compounds. This is attested by a rapidly growing number of present reports and companies specializing in this field [181–184]. Plant viral templates seem to exhibit convincing and in several aspects unique capacities for the immobilization and stabilization of bioactive peptides and proteins such as antibodies and enzymes, as illustrated above. Hence, the newly available rapid transient production methods in plants used as bioreactors [181] may be expected to promote the integration of viral nanocarriers in diagnostic systems and biosensor devices. Among those, the rigid TMV rods excel in their stable adjustable shape and durability. After simple conjugation of biotin linkers, they could be equipped with commercially available, fully active enzyme preparations at so far unsurpassed surface densities; their performance was even stabilized over weeks. Antibodies were installed with high efficiencies on TMV backbones as well, by protein A-exposing CP variants or suitable affinity tags. In conjunction with a plentitude of protocols established for the deposition of TMV on technical surfaces, it is thus likely that TMV will take over routine jobs in appropriate layouts in the longer run, and continue to forge ahead in encouraging novel concepts in biodetection, catalysis, electronics and further applications.
